# Synthesis and Photophysical Properties of the 2-(3-(2-Alkyl-6,8-diaryl-4-oxo-1,2,3,4-tetrahydroquinazolin-2-yl)propyl)-6,8-diarylquinazolin-4(3*H*)-ones

**DOI:** 10.3390/molecules19079712

**Published:** 2014-07-08

**Authors:** Mmakwena M. Mmonwa, Malose J. Mphahlele, Morad M. El-Hendawy, Ahmed M. El-Nahas, Nobuaki Koga

**Affiliations:** 1 Department of Chemistry, College of Science, Engineering and Technology, University of South Africa, P.O. Box 392, Pretoria 0003, South Africa; E-Mail: mmonwmm@unisa.ac.za; 2 Chemistry Department, Faculty of Science, Kafrelsheikh University, Kafrelsheikh 33516, Egypt; E-Mail: Morad.elhendawy@yahoo.com; 3 Chemistry Department, Faculty of Science, El-Menoufia University, Shebin El-Kom 32512, Egypt; E-Mail: amelnahas@hotmail.com; 4 Graduate School of Information Science, Nagoya University, Furo-cho, Chikusa-ku, Nagoya 464-8601, Japan; E-Mail: koga@is.nagoya-u.ac.jp

**Keywords:** tetrabromobisquinazolinones, Suzuki-Miyaura cross-coupling, tetraarylbisquinazolinones, photophysical properties

## Abstract

Iodine-catalyzed condensation of 2-amino-3,5-dibromobenzamide with cyclohexane-1,3-dione derivatives in refluxing toluene afforded the corresponding bisquinazolinones. Suzuki-Miyaura cross-coupling of the latter with arylboronic acids afforded tetraarylbisquinazolinones. The electronic absorption and emission properties of these tetraarylbisquinazolinones were measured in dimethylsulfoxide (DMSO) and acetic acid by means of UV-Vis and fluorescence spectroscopic techniques in conjunction with quantum chemical methods to understand the influence of substituents on intramolecular charge transfer (ICT).

## 1. Introduction

With their rich biological activities [1,2,3,4,5] and photophysical (absorption and luminescence) properties [6,7,8], the synthesis of 4-quinazolinone–based compounds has been the target of a great deal of research. Moreover, a three layered greenish-yellow electroluminescent device based on 2-[4ꞌ-(*N,N-*dimethylaminophenyl)]-2,3-dihydroquinazolin-4(1*H*)-one as an emissive layer sandwiched between a hole transporting layer, *N,N′*-diphenyl-*N,N′*-bis(3-methylphenyl)-1,1-biphenyl-4,4'-diamine (TPD), and an electron transporting layer, tris(8-hydroxyquinolinato)aluminium (Alq3) {ITO/TPD/MAPQ/Alq3/Al} has been developed [9]. Efforts are now being made to combine two quinazolinone moieties directly [10,11,12] and through a rigid aromatic ring[12,13] or flexible aliphatic spacer [13,14] in order to modify or enhance the biological and/or photophysical (absorption or photoluminescence) properties of the ligands. Lu *et al.* recently described a novel method which involves iodine-mediated reaction of 2-aminobenzamide with cyclohexane-1,3-dione derivatives in toluene at 110 °C to afford series of bisquinazolinones [14]. Our continued interest in the synthesis and photophysical property studies of nitrogen-containing heterocycles [15], prompted us to extend this approach to the synthesis of tetrabromo-substituted bisquinazolinones by condensing 2-amino-3,5-dibromobenzamide with cyclohexane-1,3-dione derivatives. We envisioned that the resultant tetrabromobisquinazolinones could undergo palladium-catalyzed Suzuki-Miyaura cross-coupling with arylboronic acids to afford novel polyaryl-substituted bisquinazolinones linked by a flexible aliphatic spacer to comprise donor-π-acceptor systems. The main aim was to understand the influence of substituents on intramolecular charge transfer (ICT) of these polyaryl-substituted bisquinazolinones. Consequently, the electronic absorption and photoluminescence properties of the prepared polycarbo-substituted bisquinazolinones were probed in solvents of different polarity (dimethylsulfoxide and acetic acid) by means of UV-Vis and fluorescence techniques as well as quantum chemical modelling.

## 2. Results and Discussion

### 2.1. Synthesis of 2-(3-(2-Alkyl-6,8-dibromo-4-oxo-1,2,3,4-tetrahydroquinazolin-2-yl)propyl)-6,8-dibromoquinazolin-4(3H)-ones

**Scheme 1:**
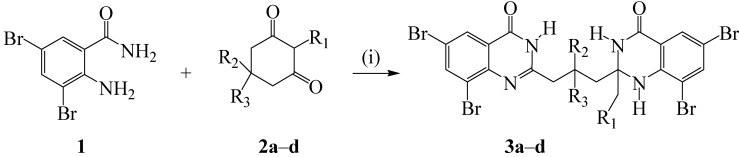
Synthesis of the 2-aryl-6,8-dibromo-2,3-dihydroquinazolin-4(1*H*)-ones **3a**–**d**.

2-Amino-3,5-dibromobenzamide (**1**) used as substrate in this investigation is readily accessible from the commercially available 2-anthranilamide and *N*-bromosuccinimide (2.5 equiv.) in chloroform-carbon tetrachloride mixture (3/2, v/v) [16]. In this investigation, we found acetic acid worked well as a solvent at room temperature in the presence of NBS to produce upon aqueous work-up and recrystallization comparable yields to those obtained using CCl_4_–CHCl_3_ mixture. Compound **1** (2.2 equiv.) was, in turn, condensed with cyclohexane-1,3-dione derivatives (1 equiv.) in the presence of iodine (0.2 equiv.) in toluene at 110 °C following literature conditions [14] to afford novel 2-(3-(2-alkyl-6,8-dibromo-4-oxo-1,2,3,4-tetrahydroquinazolin-2-yl)propyl)-6,8-dibromoquinazolin-4(3*H*)-ones **3a**–**d** (Scheme 1). The structures of the prepared products were characterized using a combination of NMR and IR spectroscopic techniques as well as mass spectrometry.

**Table:** 

	R_1_		R_2_		R_3_		% Yield 3	
**3a**	H		H		H		85		
**3b**	-CH_3_		H		H		60		
**3c**	H		-CH_3_		H		83		
**3d**	H		-CH_3_		-CH_3_		63		

*Reagents and conditions*: (i) I_2_ (cat.), toluene, reflux, 12 h.

Access to facile and efficient methods for the synthesis of halogenated heterocycles coupled with their ease of structural elaboration via metal catalyzed cross-coupling reactions make it possible to realize simple novel fluorophores. With the bisquinazolinones **3a**–**d** in hand, we next focused our attention on their reactivity in Pd-catalyzed Suzuki-Miyaura cross-coupling reactions with arylboronic acids to prepare polyaryl-substituted bisquinazolinones.

### 2.2. Suzuki-Miyaura Cross-coupling of the 2-(3-(2-Alkyl-6,8-dibromo-4-oxo-1,2,3,4-tetrahydroquinazolin-2-yl)propyl)-6,8-dibromoquinazolin-4(3H)-ones

**Scheme 2:**
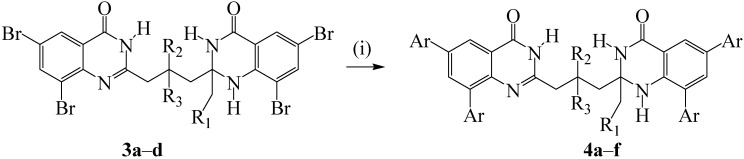
Palladium-catalyzed Suzuki-Miyaura cross-coupling of **3a**–**d** with arylboronic acids.

While it is possible to control the regioselectivity of Suzuki-Miyaura cross-couplings for dihaloheterocycles bearing different halogen atoms (I *vs.* Br/Cl or Br *vs.* Cl), it is relatively difficult to achieve high levels of regioselectivity in the case involving derivatives bearing similar halogen atoms. Computed bond dissociation energies at B3LYP and G3B3 levels reveal that all of the positions on the fused benzo ring of various heterocycles bearing identical halogen atoms have comparable C-X bond dissociation energies [17]. The most common outcome in Suzuki-Miyaura cross-coupling reactions involving dihaloaromatic precursors bearing similar halogen atoms is exhaustive multiple coupling and an excess of the arylboronic acid or ester coupling partner is often employed to drive the reaction to completion [18]. Reduced reaction time and complete conversion of the substrate to the products are usually observed in the presence of alkylphosphine ligands, which are known to coordinate with palladium and increase its electron density more so than arylphosphines and, in turn, accelerate the oxidative addition and reductive elimination steps in the catalytic cycle [19,20,21,22]. Based on the literature precedents, we subjected compounds **3a**–**d** to the Suzuki-Miyaura cross-coupling with excess arylboronic acids (4.5 equiv.) using dichlorobis(triphenylphosphine)palladium(II)-tricyclohexylphosphine catalyst complex (PdCl_2_(PPh_3_)_2_-PCy_3_) in dioxane-water (3/1, v/v) in the presence of K_2_CO_3_ as a base (Scheme 2). We isolated the corresponding tetraarylbisquinazolinones **4a**–**l** in high purity and reasonable yields without the need for column chromatographic separations. Their structures were characterized using a combination of NMR and IR spectroscopic techniques and their accurate calculated *m/z* values represent a closest fit consistent with the incorporation of four aryl moieties. The polycarbo-substituted bisquinazolinones bearing aryl substituents on the fused benzo ring cannot be easily prepared using conventional methods for the synthesis of bisquinazolinones.

**Table:** 

	R_1_		R_2_		R_3_		Ar		% Yield	
**4a**	H		H		H		-C_6_H_5_		80
**4b**	H		H		H		4-FC_6_H_4_-		81
**4c**	H		H		H		4-MeOC_6_H_4_-		74
**4d**	-CH_3_		H		H		-C_6_H_5_		63
**4e**	-CH_3_		H		H		4-FC_6_H_4_-		63
**4f**	-CH_3_		H		H		4-MeOC_6_H_4_-		60
**4g**	H		-CH_3_		H		-C_6_H_5_		75
**4h**	H		-CH_3_		H		4-FC_6_H_4_-		70
**4i**	H		-CH_3_		H		4-MeOC_6_H_4_-		73
**4j**	H		-CH_3_		-CH_3_		-C_6_H_5_		76
**4k**	H		-CH_3_		-CH_3_		4-FC_6_H_4_-		77
**4l**	H		-CH_3_		-CH_3_		4-MeOC_6_H_4_-		81

*Reagents and conditions*: (i) ArB(OH)_2_ (4.5 equiv.), PdCl_2_(PPh_3_)_2_, PCy_3_, K_2_CO_3_, dioxane-water (3/1, v/v), reflux, 5 h.

Polysubstituted bisquinazolinones **4a**–**l** comprise an electron-deficient quinolin-4(1*H*)-one and quinolin-4(3*H*)-one moieties as electron-acceptors each linked directly to the aryl groups at the 6- and 8-position to comprise a donor-π-acceptor system. As a prelude to bisquinazolinones with photophysical properties, we determined the absorption and fluorescence properties of compounds **3a**–**d** and **4a**–**l** in polar aprotic dimethyl sulfoxide (DMSO) and polar protic acetic acid (AcOH). These solvents were chosen due to poor or lack of solubility of these compounds in various solvents.

### 2.3. Photophysical Property Studies of Compounds **4a**–**l**

To understand the influence of substituents on intramolecular charge transfer (ICT), which occurs through-space interaction or orbital overlap between donor and acceptor groups [23,24], we measured the absorption and emission spectra for the polysubstituted bisquinazolinone derivatives **4a**–**l** in selected solvents of different polarity in combination with quantum chemical techniques. To our knowledge, no photophysical property studies of bisquinazolinones has been reported before. Moreover, there is no literature precedents for the photophysical properties of the 2-susbtituted quinazolin-4-ones bearing aryl susbtituents on the fused benzo ring.

#### 2.3.1. UV-Vis Absorption Properties of the 2-(3-(2-Alkyl-6,8-diaryl-4-oxo-1,2,3,4-tetrahydroquinazolin-2-yl)propyl)-6,8-diarylquinazolin-4(3*H*)-ones **4a**–**l**

**Figure 1:**
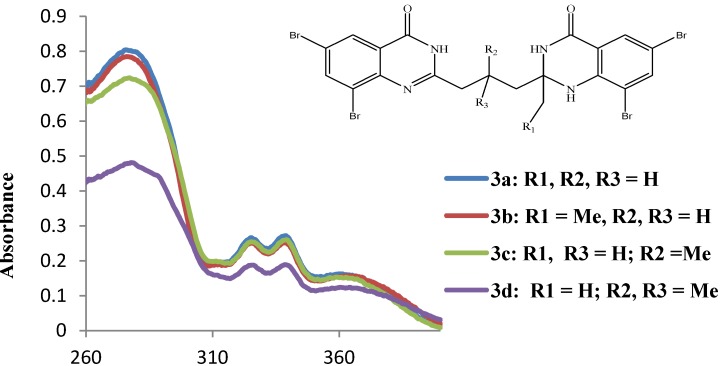
UV-Vis Spectra of **4a**–**c** in DMSO at rt (conc. = 1.0 × 10^−5^ mol/L).

We first measured the electronic absorption spectra of compounds **3a**–**d** as DMSO solutions at room temperature and they reveal the presence of four absorption bands of different intensities in the ultraviolet region (260–400 nm) at λ_max_ *ca.* 278, 325, 339 and 360 nm, respectively (Figure 1, Table 1). The highest intensity absorption bands for these compounds, which resonate at λ_max_ *ca.* 278 nm and are due to the π–π* transition of the quinazolinone backbones. The trend in intensity of the absorption maxima seems to be influenced by the substitution pattern on the propyl chain and the 2-position of the quinazolin-4(1*H*)-one moiety and the trend in molar extinction coefficients is as follows: **3a** > **3b** > **3c** > **3d** (Table 1). The two absorption bands at λ_max_ *ca*. 325 and 339 nm of equal intensity are presumably due to the intramolecular charge transfer of the heterocyclic rings, *i.e.*, from the fused benzo ring and N=C-N fragments to the carbonyl group and the n–π* transition of the carbonyl moiety [8]. These bands and the difference in their absorption wavelengths reflect the influence of bromine atoms on the 4(1*H*)-oxo and 4(3*H*)-oxo moieties on the π–π* and n–π* transition in analogy with literature precedent for the mesoionic 2-(2-dialkylamino)-1,3-dithiol-2-ylium-4-yl)phenolates bearing two bromine substituents on the phenolate moiety [25]. The relatively weak broad band centered at λ_max_ *ca*. 360 nm of charge transfer nature may be the result of weakly allowed π–π* transitions and/or quinazolinone-based n–π* transition of the electron-rich heteroatoms.

**Table 1:** The absorption data for compounds **3a**–**d**.

Compound	λ_max_ (nm)	Molar Extinction Coefficient
	DMSO	(ε) × 10^4^ mol^−1^ cm^−1^
**3a**	275.8, 325.3, 338.8, 360.4	1.6 , 0.5, 0.5, 0.3
**3b**	276.1, 325.3, 338.8, 362.2	1.5, 0.5, 0.5, 0.3
**3c**	277.0, 325.3, 339.1, 359.5	1.4, 0.5, 0.5, 0.3
**3d**	277.9, 325.9, 338.8, 360.4	1.0, 0.4, 0.4, 0.2

**Figure 2:**
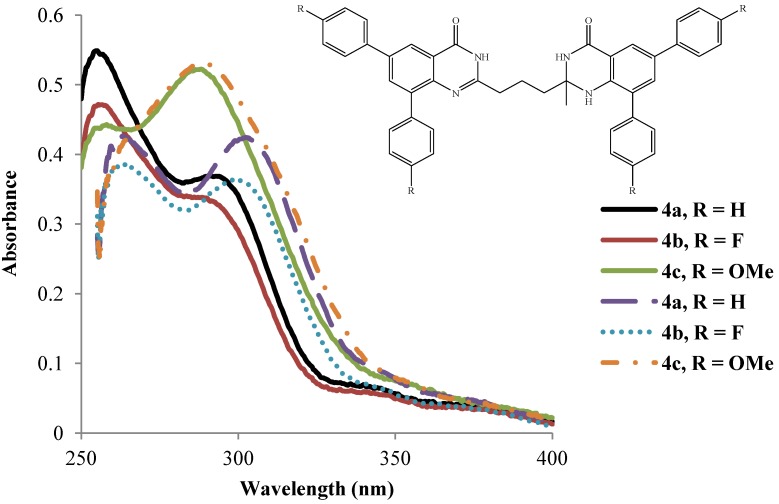
UV-Vis spectra of **4a**–**c** in DMSO (dashed lines) and CH_3_CO_2_H (solid lines) at rt (conc. = 1.0 × 10^−5^ mol/L).

**Figure 3:**
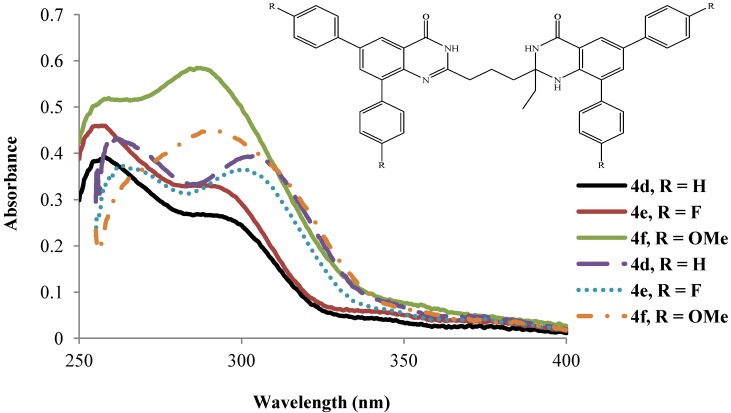
UV-Vis spectra of **4d**–**f** in DMSO (dashed lines) and CH_3_CO_2_H (solid lines) at rt (conc. = 1.0 × 10^−5^ mol/L).

**Figure 4:**
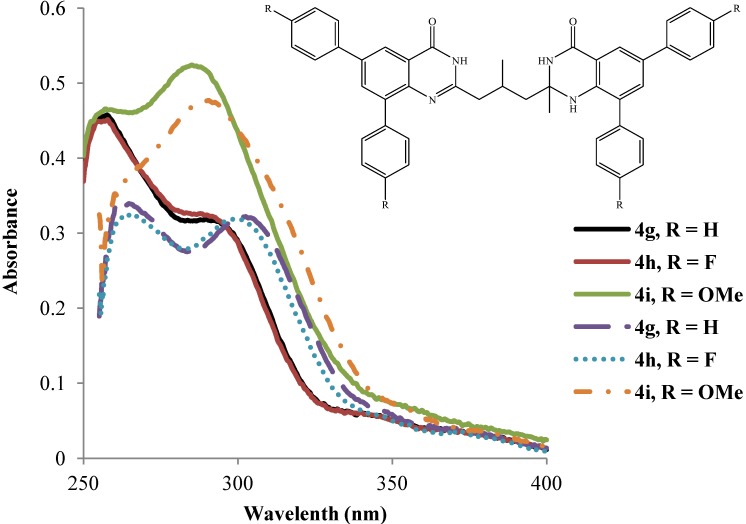
UV-Vis spectra of **4g**–**i** in DMSO (dashed lines) and CH_3_CO_2_H (solid lines) at rt (conc. = 1.0 × 10^−5^ mol/L).

**Figure 5:**
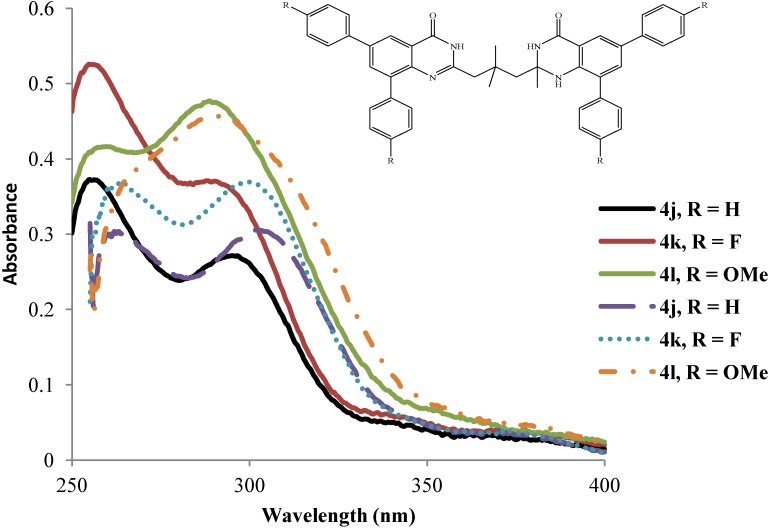
UV-Vis spectra of **4j**–**l** in DMSO (dashed lines) and CH_3_CO_2_H (solid lines) at rt (conc. = 1.0 × 10^−5^ mol/L).

The absorption spectra of the polyaryl-substituted bisquinazolinones **4a**–**l** (Figure 2, Figure 3, Figure 4, Figure 5 and Table 2) were acquired in DMSO (dashed lines) and acetic acid (solid lines) at room temperature and are characterized by two intense absorption maxima in the ultraviolet region. The two absorption maxima of comparable intensity in the regions λ_max_ 250–270 nm and λ_max_ 280–340 nm in DMSO and λ_max_ 253–258 nm and 288–295 nm in acetic acid are well resolved for the phenyl- and 4-fluorophenyl-substituted derivatives in both solvents (Figure 2, Figure 3, Figure 4 and Figure 5). A distinguishing feature for the UV-Vis spectra of the 4-methoxyphenyl–substituted derivatives **4c**, **4f**, **4i** and **4l** in DMSO is that the two bands overlap to yield a high intensity single broad band at λ_max_ 290–294 nm with a poorly defined shoulder in the low wavelength region. The broad bands for the methoxyphenyl–substituted derivatives observed in DMSO are however resolved into two distinct broad maxima each at λ_max_ *ca.* 259 and 289 nm in the polar protic acetic acid. The shorter wavelength bands in the region λ_max_ 250–270 nm for compounds **4a**–**l** are ascribed to the superimposition of the π–π* transition of the fused benzo rings and the n–π* transition of nitrogen atoms as well as intramolecular donor-acceptor charge transfer absorption of the aryl substituents in analogy with the literature assignment for the quinazolin-4(3*H*)-ones [26]. The longer wavelength bands in the region λ_max_ 280–340 nm are due to the intramolecular charge transfer of the heterocyclic rings, *i.e.*, from the fused benzo ring and N=C-N fragments to the carbonyl group and the n–π* transition of the carbonyl moiety [8]. The corresponding long wavelength band in the case of the corresponding precursors **3a**–**d** have been found to comprise two separate bands of equal intensity, which are red shifted by the presence of the two bromine atoms on the 4(1*H*)-oxo and 4(3*H*)-oxo moieties (see Figure 1). The overlap of the short and long wavelength bands in DMSO in the case of the 4-methoxyphenyl–substituted derivatives **4c**, **4f**, **4i** and **4l** presumably results from an additional intramolecular charge transfer (ICT) due to the increased propensity of the methoxy groups for the lone pair electron delocalization. Hydrogen bonding between the strongly polar protic acetic acid and nitrogen atoms and the carbonyl groups of the heterocyclic rings of compounds **4c**, **4f**, **4i** and **4l** probably leads to well-resolved primary and secondary absorption maxima of increased intensities. This strong intermolecular hydrogen bonds would make the bisquinazolinone framework more electron deficient and result in the observed reduced intensity of the longer wavelength band, which is more pronounced for the phenyl- (**4a**, **4d**, **4g** and **4j**) and 4-fluorophenyl substituted derivatives (**4b**, **4e**, **4h** and **4k**). Increased intensities for the longer wavelength maxima for the methoxyphenyl–substituted derivatives (**4c**, **4f**, **4i** and **4l**), on the other hand, probably result from increased ICT from the electron-rich methoxyphenyl substituted benzo rings and N=C-N fragments to the carbonyl group.

Within each series, the nature of the substituent on the aryl groups and the size of the substituent on the 2-position of the quinazolin-4(1*H*)-one moiety as well as the substitution pattern on the propyl chain seem to influence the relative intensity and the wavelengths of the absorption maxima in both solvents. The intensities of both absorption bands in acetic acid (solid lines) for compounds **4a**–**c** bearing a 2-methyl substituent on the quinazolin-4(1*H*)-one fragment reflect the electron donating effect of 4-R on the aryl groups **4a** (H) < **4b** (F) < **4c** (OMe) (Figure 2). These compounds exhibit comparable absorption wavelengths for the primary band with the following trend in wavelengths for the secondary band:**4c** (OMe) < **4b** (F) < **4a** (H). Although the primary absorption maximum of **4c** comprises a poorly resolved shoulder in DMSO (dashed lines), it is in the same wavelength region as those for **4a** and **4b** (Figure 2). The trend in intensities of the primary and secondary absorption bands for compounds **4a**–**c** is as follows **4c** > **4a** > **4b** with the following trend in absorption wavelengths **4c** < **4b** < **4a**.

A relatively bulky ethyl group on the heterocyclic ring of compounds **4d**–**f** in acetic acid, on the other hand, has less effect on the absorption wavelengths of the primary and secondary bands (Figure 3). The intensity of their absorption maxima, however, reflects the electron donating effect of the substituent on the aryl rings: **4d** (H) < **4e** (F) < **4f** (OMe). Similar trends in intensity of the absorption maxima, but different absorption wavelengths are observed for compounds **4d**–**f** in DMSO. The absorption wavelength values for compounds **4d** and **4e** are comparable and red shifted than for the 4-methoxyphenyl–substituted derivative **4f**.

Both primary and secondary maxima for compounds **4g** and **4h** bearing 2-methyl group on the quinazolin-4(1*H*)-one and a methyl group on the propyl chain absorb in the same wavelength regions in DMSO (Figure 4). The primary band for compound **4i** comprises a poor shoulder in the same wavelength region as for compounds **4g** and **4h** and the following trend in intensity is observed: **4h** < **4g** < **4i**. The intensities for the secondary bands for compounds **4g** and **4h** in DMSO are comparable and less than for **4i**, which resonates at significantly lower wavelength (Figure 4). The wavelengths and the intensities of the primary absorption bands for compounds **4g**–**i** in acetic acid are comparable and their secondary bands also resonate in the same wavelength region with high intensity for **4i** than for **4g** and **4h** (Figure 4).

For compounds **4j**–**l** bearing two methyl groups on the propyl chain and the 2-methyl group on the quinazolin-4(1*H*)-one framework, the intensities of the primary and secondary absorption maxima in DMSO show the following trend: **4j** < **4k** < **4l** with reversed trend in wavelengths **4l** < **4k** < **4j** (Figure 5). A similar trend in intensity of the absorption maxima with comparable wavelengths is observed for compounds **4j**–**l** in acetic acid (solid lines).

In general, the absorption bands of compounds **4a**–**l** are slightly red shifted in DMSO than in the polar protic acetic acid. The extinction coefficients are relatively high indicating that the π–π* transition is strongly allowed.

#### 2.3.2. Emission Properties of the 2-(3-(2-Alkyl-6,8-diaryl-4-oxo-1,2,3,4-tetrahydroquinazolin-2-yl)propyl)-6,8-diarylquinazolin-4(3*H*)-ones **4a**–**l**

**Figure 6:**
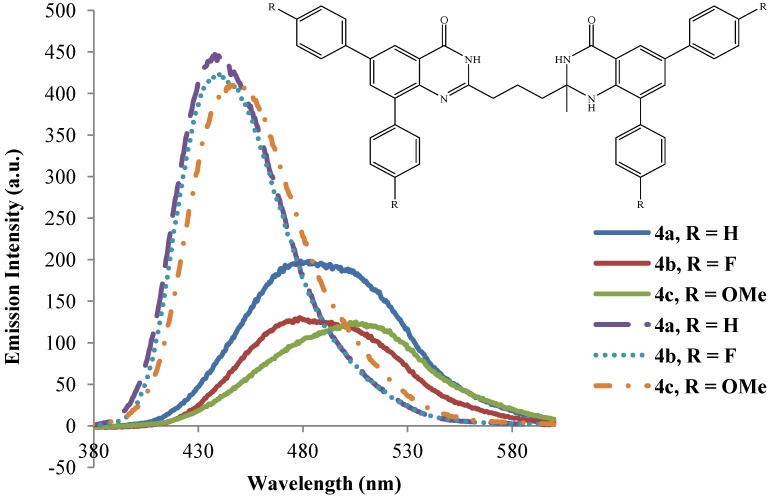
Emission spectra of **4a**–**c** (λ_ex_ = 270 nm) in DMSO (dashed lines) and CH_3_CO_2_H (solid lines) at rt (conc. = 1.0 × 10^−4^ mol/L).

**Figure 7:**
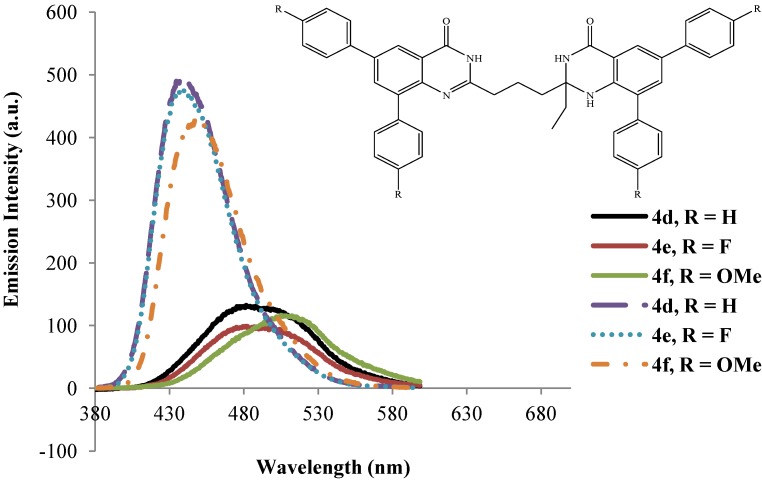
Emission spectra of **4d**–**f** (λ_ex_ = 270 nm) in DMSO (dashed lines) and CH_3_CO_2_H (solid lines) at rt (conc. = 1.0 × 10^−4^ mol/L).

**Figure 8:**
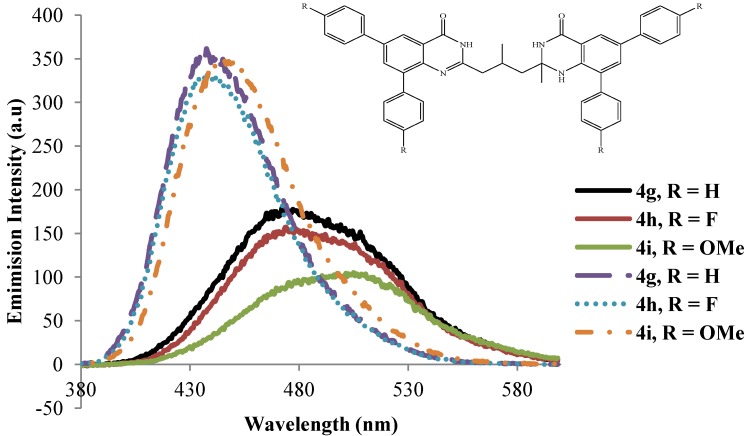
Emission spectra of **4g**–**i** (λ_ex_ = 270 nm) in DMSO (dashed lines) and CH_3_CO_2_H (solid lines) at rt (conc. = 1.0 × 10^−4^ mol/L).

**Figure 9:**
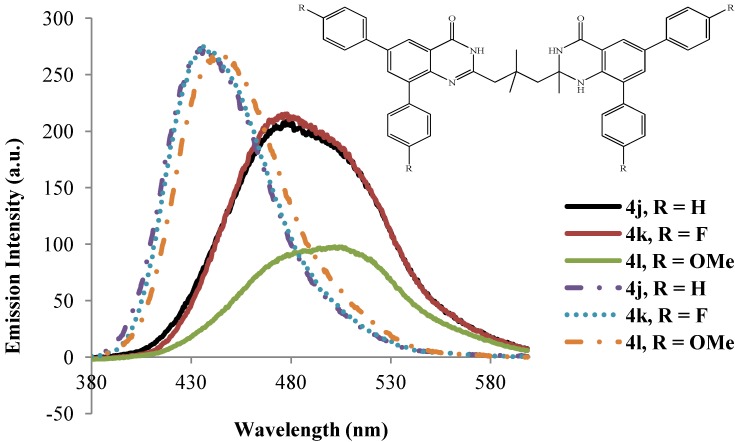
Emission spectra of **4j**–**l** (λ_ex_ = 270 nm) in DMSO (dashed lines) and CH_3_CO_2_H (solid lines) at rt (conc. = 1.0 × 10^−4^ mol/L).

The dibromo compounds **3a**–**d** are hardly emissive in the excitation wavelength 260 ± 10 nm presumably due to heavy halogen atom [27].The analogous 8-benzyloxy-5,7-dibromoquinoline were found to be hardly emissive in trifluoroacetic acid and semi-empirical calculations revealed that the S_1_ state for these dibromo derivatives is the π–σ* excited state where the LUMO is a σ* orbital localized at the C-Br bonds [18]. The emission properties of compounds **4a**–**l** have also been studied in polar aprotic DMSO (dashed lines) and strongly polar protic acetic acid (solid lines) at excitation wavelength 270 nm (Figure 6, Figure 7, Figure 8 and Figure 9). The emission spectra of these compounds in both solvents show a single emission band at room temperature in the blue to green region, λ_em_ = 430–510 nm. These bands are attributed to π–π* transition resulting from direct π-electron delocalization by the aryl groups into the quinazolinone framework. Within each series the emission wavelengths and the Stokes shift as well as the quantum yield values are influenced by the nature of the substituent on the *para* position of the 6- and 8-aryl groups, the size of the substituent on the 2-position of the quinazolin-4(1*H*)-one moiety and the substitution pattern on the propyl chain (Table 2). Generally, the polarity of a solvent is known to influence the emission spectra of fluorophores by changes in quantum yields and spectral shifts and the fluorescent probe is weakly fluorescent in more polar solvents [28]. Likewise, the π–π* state is more polarizable than the ground state and this causes measurable displacements of the π–π* transition towards the red bands [29]. The polarities of acetic acid and DMSO are 0.648 and 0.444, respectively [30], and the strongly polar protic acetic acid causes measurable displacements of the π–π* transitions of compounds **4** towards the red bands. Moreover, strong hydrogen bonding between the solute and acetic acid leads to reduced intensity and increased broadening of the photoluminescence bands. Considerable red-shift of the fluorescence maxima and the increase of the Stokes shifts as well as the emission bandwidths with increasing solvent polarity point to the charge transfer character of the solvent-equilibrated fluorescent charge transfer states of these molecules.

The presence of the strongly electron-donating 4-methoxyphenyl groups generally leads to slight shifts of the emission wavelength to the red in DMSO presumably due to increased π–π* and n–π* transitions. The intensities of the emission bands for these compounds in acetic acid are however reduced presumably due to additional hydrogen bonding with the methoxy groups, which in our view would weaken their propensity for electron donation into the aromatic rings. The solvent-dependent emission characteristics of compounds **4a**–**l** which result from the dipolar interaction with the polar solvent suggest the ICT character of the emission state, in which the HOMOs and LUMOs are presumably localized on the aryl rings and the quinazolinone-based moieties, respectively. To proof this hypothesis, we used computational methods to determine the HOMO and LUMO orbital energies of the bisquinazolinone derivatives.

**Table 2:** The absorption and emission data for compounds **4a**–**l**.

4	λ_max_ (nm)	(ε) × 10^4^	λ_max_ (nm)	(ε) × 10^4^	λ_em_ (nm)	λ_em_ (nm)	^(a)^ Quantum	^(a)^ Quantum	Stokes Shift	Stokes Shift
	DMSO	Mol^−1^cm^−1^	AcOH	Mol^−1^cm^−1^	DMSO	AcOH	Yields (Φ) × 10^−3^	yields (Φ) × 10^−3^		
							DMSO	AcOH	DMSO	AcOH
**4a**	263.2	4.2	255.1	5.5	438.0	479.0	7.5	2.6	174.8	223.9
	302.5	4.2	292.3	3.7	438.0	479.0	7.5	3.8	135.5	186.7
**4b**	263.2	3.9	255.4	4.7	439.0	478.5	7.8	2.0	175.8	223.1
	300.1	3.6	283.6	3.4	439.0	478.5	8.3	2.7	138.9	194.9
**4c**	291.4	5.3	258.1	4.4	448.0	505.5	5.5	2.0	156.6	247.4
			288.1	5.2		505.5		1.7		217.4
**4d**	262.0	4.3	257.8	3.9	439.0	481.5	8.2	2.4	177.0	223.7
	303.4	3.9	293.8	2.6	439.0	481.5	9.0	3.6	135.6	187.7
**4e**	261.7	3.6	257.5	4.6	439.0	484.5	9.1	1.5	177.3	227.0
	302.2	3.6	291.4	3.3	439.0	484.5	9.3	2.1	136.8	193.1
**4f**	291.4	4.5	259.0	5.2	447.5	505.0	6.8	1.6	156.1	246.0
			286.3	5.8		505.0		1.4		218.7
**4g**	263.2	3.4	257.5	4.6	437.5	475.5	8.0	2.8	174.3	218.0
	302.5	3.2	292.3	3.2	437.5	475.5	8.4	4.1	135.0	183.2
**4h**	263.5	3.3	257.8	4.5	437.0	476.5	7.7	2.4	173.5	218.7
	300.4	3.2	288.4	3.3	437.0	476.5	7.8	3.3	136.6	188.1
**4i**	290.5	4.8	257.2	4.7	443.0	504.0	5.4	1.6	152.5	246.8
			285.1	5.2		504.0		1.5		218.9
**4j**	262.0	3.0	254.8	3.7	434.5	477.5	6.4	4.0	172.5	222.7
	304.0	3.0	295.3	2.7	434.5	477.5	6.4	5.5	130.5	182.2
**4k**	262.6	3.7	254.8	5.2	436.0	477.0	5.3	2.9	173.4	222.2
	300.1	3.7	288.1	3.7	436.0	477.0	5.3	4.2	135.9	188.9
**4l**	293.3	4.6	259.6	4.2	447.5	502.0	4.2	1.7	154.2	242.4
			288.7	4.8		502.0		1.5		213.3

^(a)^ The relative quantum yields were calculated according to the equation indicated under Experimental section using quinine sulfate (emission range 400–600 nm) as the standard (Φq = 0.55) in 1.0 N H_2_SO_4_ [31].

### 2.4. Quantum Chemical Calculations

**Figure 10:**
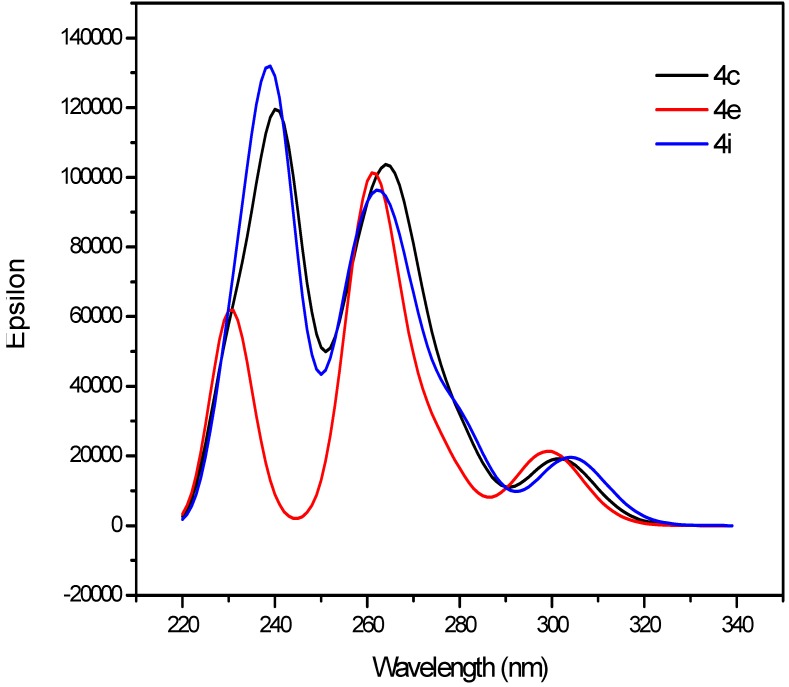
Computed absorption spectra of **4c**, **4e** and **4i**.

**Figure 11:**
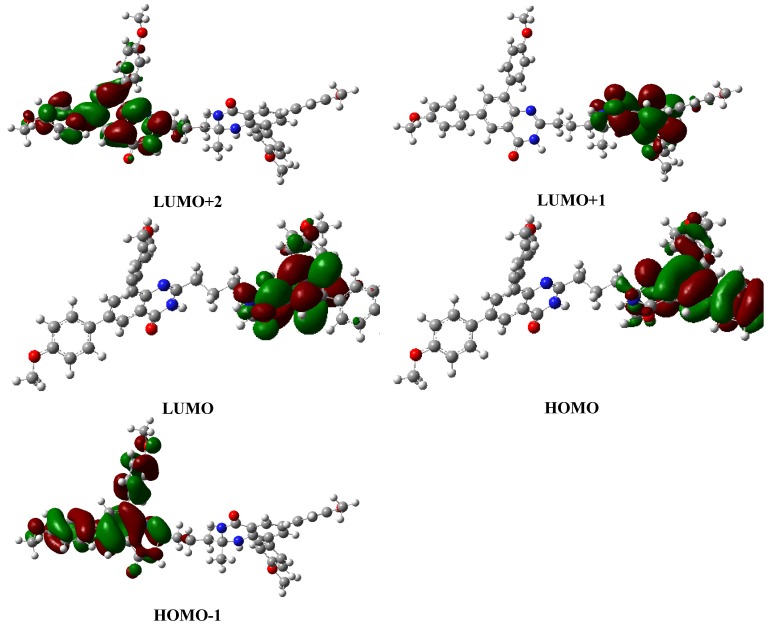
Orbital topology for **4c** as representative compound.

To further establish the structural features and molecular orbitals of the bisquinazolinones **4**, we carried out a theoretical approach using density functional theory at the LC-BLYP/6-31+G(d,p) [32] level as implemented in Gaussian 09 suite [33]. The geometries were optimized at the LC-BLYP/6-31G(d,p) level in DMSO to obtain reasonable structures for the subsequent electronic structure computations. The bulk solvent effects were included *via* the Polarisable Continuum Model (PCM), using the integral equation formalism variant (IEFPCM) [34] in DMSO. The computed absorption spectra of representative examples **4c**, **4e** and **4i** in DMSO were reproduced at LC-BLYP level with three absorption peaks at λ *ca.* 235, 260 and 300 nm (Figure 10). A good agreement was found between the experimental and computed wavelength maxima with an average error of ± 3 nm (Table 3). The charge transfer band at λ *ca.* 300 nm results mainly (60%) due to an electronic transition from HOMO to LUMO+2. A smaller contribution (*ca.* 20%) to this state is attributed to an electronic transition from HOMO-4 to LUMO+2. The HOMO, on the other hand, is mainly localized on one of quinazolinonyl subunits (4(1*H*)-oxo), the electron density of LUMO transferred completely to the left hand side (4(3*H*)-oxo) as shown in Figure 11. This electronic transition is attributed to the intramolecular charge transfer band. The absorption band at λ *ca.* 260 nm is due to two electronic transitions- one from HOMO-1 to LUMO+1 (50%) and the other from HOMO-1 to LUMO (33%). The electron density of LUMO and LUMO+1 are mainly localized on the heterocyclic ring of quinazolin-4(1*H*)-one framework. However, electron density of HOMO-1 is mainly localized on the diarylquinazolin-4(3*H*)-one moiety. The n–π* transition overlapped with π–π* transition due to high conjugation on both sides of the aliphatic spacer. No significant changes were observed in the electron density distribution of the investigated compounds due to the aliphatic spacers. Inspection of the computed ground- and excited-state dipole moments using LC-BLYP functional confirms the first singlet excited state is somewhat of more polar character than the ground state (Table 3). The dipole moments of the excited state are slightly larger than those of the ground state by one debye. Similar results were obtained for these compounds presumably due to the highly symmetric molecular structure.

**Table 3:** The computed dipole moments and absorption spectra of compounds **4a**–**l**.

Compound	D_ground (Debye)_	D_excited (Debye)_	λ_max (nm)_
**4a**	7.66	8.24	260, 299
**4b**	7.45	8.37	261, 300
**4c**	5.96	5.44	266, 301
**4d**	7.70	8.09	261, 299
**4e**	8.19	8.33	262, 299
**4f**	6.35	7.41	266, 301
**4g**	5.71	6.62	262, 301
**4h**	9.84	9.92	261, 300
**4i**	8.99	9.02	260, 304
**4j**	5.62	6.07	262, 299
**4k**	6.45	6.47	262, 298
**4l**	5.81	5.94	260, 297

## 3. Experimental

### 3.1. General Information

Melting points were recorded on a Thermocouple digital melting point apparatus and are uncorrected. IR spectra were recorded as powders using a Bruker VERTEX 70 FT-IR Spectrometer with a diamond ATR (attenuated total reflectance) accessory by using the thin-film method. For column chromatography, Merck kieselgel 60 (0.063–0.200 mm) was used as stationary phase. The UV-Vis spectra were recorded on a Cecil CE 9500 (9000 Series) UV-Vis spectrometer while emission spectra were taken using a Perkin Elmer LS 55 fluorescence spectrometer. The quantum efficiencies of fluorescence (Φ_fl_) were obtained with the following equation:

Φ_x_ = Φ_st_*(*F_x_*/*F_st_*)*(A_st_/A_x_)*(*n*_x_^2^/*n*_st_^2^)

where *F* denotes the area under the fluorescence band (*F* = ^a^*I*_fl_(λ), where *I*_fl_(λ) is the fluorescence intensity at each emission wavelength), A denotes the absorbance at the excitation wavelength, and *n* is the refractive index of the solvent [35]. NMR spectra were obtained as DMSO-*d*_6_ solutions using Varian Mercury 300 MHz NMR spectrometer and the chemical shifts are quoted relative to the solvent peaks. Low- and high-resolution mass spectra were recorded at the University of Stellenbosch Mass Spectrometry Unit using Synapt G2 Quadrupole Time-of-flight mass spectrometer.

### 3.2. Synthesis of 3,5-Dibromobenzamide (**1**)

A mixture of 2-aminobenzamide/anthranilimide (10.00 g, 74.10 mmol) and *N*-bromosuccinimide (26.37 g, 148.20 mmol) in acetic acid (400 mL) was stirred at room temperature for 3 h. The mixture was quenched with an ice-cold water and the resulting precipitate was filtered dry on a sintered funnel, washed thoroughly with an ice-cold ethanol and then dried in an oven to afford **(1)** (17.00 g, 78%), mp. 213–215 °C (216–217 °C [16]).

### 3.3. Typical Procedure for the Preparation of the 2-(3-(2-Alkyl-6,8-dibromo-4-oxo-1,2,3,4-tetrahydroquinazolin-2-yl)-propyl)-6,8-dibromoquinazolin-4(3H)-ones **3a**–**d**

A stirred mixture of 2-amino-3,5-dibromobenzamide (**1**, 2.2 equiv.), cyclohexane-1,3-dione derivative **2** (1.0 equiv.) and iodine (10% of **1**) in toluene (15 mL/mmol of **2**) was heated at 110 °C for appropriate time. The mixture was allowed to cool to room temperature and the solvent was evaporated under reduced pressure. The resulting precipitate was filtered and then washed sequentially with water and warm ethanol. The product was further dried in an oven to afford **3** as a solid. The following products were prepared in this fashion:

*2-(3-(6,8-Dibromo-2-methyl-4-oxo-1,2,3,4-tetrahydroquinazolin-2-yl)propyl)-6,8-dibromoquinazolin-4(3H)-ones* (**3a**). A mixture of **1** (1.10 g, 3.74 mmol), cyclohexane-1,3-dione (**2a**, 0.19 g, 1.70 mmol) and iodine (0.13 g, 0.51 mmol) in toluene (25.00 mL) was refluxed for 5 h to afford **3a** as a solid (0.96 g, 85%), mp. 286‒290 °C; ν_max_ (neat) cm^−1^ 3379, 3196, 3056, 1669, 1600, 1464, 1386, 1253, 1075, 897, 808, 760, 697, 619; δ_H_ (300 MHz, DMSO-*d*_6_) 1.41 (s, 3H), 1.67–1.91 (m, 4H), 2.46–2.55 (m, 2H), 6.17 (s, 1H), 7.61 (d, *J* 3.0 Hz, 1H), 7.64 (d, *J* 3.0 Hz, 1H), 8.08 (d, *J* 3.0 Hz, 1H), 8.21 (d, *J* 3.0 Hz, 1H), 8.32 (s, 1H), 12.51 (s, 1H); δ_C_ (75 MHz, DMSO-*d*_6_) 21.8, 28.3 (2 × C), 34.8, 70.2, 107.6, 108.7, 117.2, 118.5, 123.5, 123.9, 128.1, 129.6, 138.2, 139.8, 143.8, 145.9, 159.4, 160.8, 161.2; *m/z* 665 (100, MH^+^); HRMS (ES): MH^+^, found 664.8846. C_20_H_17_N_4_O_2_^79^Br_4_^+^ requires 664.9826.

*2-(3-(6,8-Dibromo-2-ethyl-4-oxo-1,2,3,4-tetrahydroquinazolin-2-yl)propyl)-6,8-dibromoquinazolin-4(3H)-ones* (**3b**). A mixture of **1** (1.10 g, 3.74 mmol), 2-methylcyclohexane-1,3-dione (**2b**, 0.22 g, 1.70 mmol) and iodine (0.13 g, 0.51 mmol) in toluene (25.00 mL) was refluxed for 12 h to afford **3b** as a solid (0.69 g, 60%), mp. 307–310 °C, ν_max_ (neat) cm^−1^ 3389, 3177, 1673, 1614, 1488, 1445, 1363, 1344, 1259, 1225, 1133, 1017, 939, 875, 737, 681, 626; δ_H_ (300 MHz, DMSO-*d*_6_) 0.83 (t, *J* 7.8 Hz, 3H), 1.63 (q, *J* 7.8 Hz, 2H), 1.57–1.72 (m, 4H), 2.56–2.60 (m, 2H), 6.04 (s, 1H), 7.63 (d, *J* 3.0 Hz, 1H), 7.68 (d, *J* 3.0 Hz, 1H), 8.13 (d, *J* 3.0 Hz, 1H), 8.21 (d, *J* 3.0 Hz, 1H), 8.28 (s, 1H), 12.55 (s, 1H); δ_C_ (75 MHz, DMSO-*d*_6_) 8.31, 21.4 (2 × C), 33.4, 34.9, 73.1, 107.0, 108.1, 116.5, 118.5, 123.5, 123.9, 128.1, 129.4, 138.2, 139.9, 144.3, 146.0, 159.4, 160.8, 161.4; *m/z* 679 (100, MH^+^); HRMS (ES): MH^+^, found 679.0086. C_21_H_19_N_4_O_2_^79^Br_4_^+^ requires 679.0092.

*2-(3-(6,8-Dibromo-2-methyl-4-oxo-1,2,3,4-tetrahydroquinazolin-2-yl)-2-methylpropyl)-6,8-dibromo-quinazolin-4(3H)-ones* (**3c**). A mixture of **1** (1.10 g, 3.74 mmol), 5,5-dimethylcyclohexane-1,3-dione (**2c**, 0.24 g, 1.70 mmol) and iodine (0.13 g, 0.51 mmol) in toluene (25.00 mL) was refluxed for 12 h to afford **3c** as a solid (0.95 g, 83%), mp. 312–314 °C; ν_max_ (neat) cm^−1^ 3389, 3177, 1673, 1614, 1597, 1560, 1488, 1445, 1363, 1344, 1225, 1133, 1017, 939, 875, 737, 681, 626; δ_H_ (300 MHz, DMSO-*d*_6_) 1.03 (d, *J* 6.3 Hz, 3H), 1.46 (s, 3H), 1.59 (dd, *J* 7.8 and 13.5 Hz, 1H), 2.07 (dd, *J* 10.8 and 13.5 Hz, 1H), 2.11‒2.51 (m, 1H), 2.39–2.55 (m, 2H), 6.22 (s, 1H), 7.31 (d, *J* 3.0 Hz, 1H), 7.59 (d, *J* 3.0 Hz, 1H), 8.13 (d, *J* 3.0 Hz, 1H), 8.24 (d, *J* 3.0 Hz, 1H), 8.33 (s, 1H), 12.51 (s, 1H); δ_C_ (75 MHz, DMSO-*d*_6_) 22.7, 28.0, 28.4, 43.2, 46.0, 70.2, 107.6, 108.4, 117.2, 118.5, 123.4, 123.8, 128.0, 129.7, 137.6, 139.9, 143.2, 145.7, 158.4, 160.8, 161.2; *m/z* 679 (100, MH^+^); HRMS (ES): MH^+^, found 679.0203. C_21_H_19_N_4_O_2_^79^Br_4_^+^ requires 679.0092.

*2-(3-(6,8-Dibromo-2-methyl-4-oxo-1,2,3,4-tetrahydroquinazolin-2-yl)-2,2-dimethylpropyl)-6,8-dibromoquinazolin-4(3H)-ones* (**3d**). A mixture of **1** (1.10 g, 3.74 mmol), 5,5-dimethylcyclohexane-1,3-dione (**2d**, 0.24 g, 1.70 mmol) and iodine (0.13 g, 0.51 mmol) in toluene (25.00 mL) was refluxed for 24 h to afford **3d** as a solid (0.74 g, 63%), mp. 315–318 °C; ν_max_ (neat) cm^−1^ 3397, 3167, 3053, 1685, 1655 1606, 1491, 1464, 1450, 1318, 1250, 1214, 1028, 894, 794, 758, 696, 642, 612; δ_H_ (300 MHz, DMSO-*d*_6_) 1.04 (s, 3H), 1.12 (s, 3H), 1.53 (s, 3H), 1.79 (d, *J* 15.3 Hz, 1H), 2.27 (d, *J* 15.3 Hz, 1H), 2.66 (d, *J* 13.8 Hz, 1H), 3.03 (d, *J* 13.8 Hz, 1H), 6.66 (s, 1H), 7.64 (d, *J* 3.0 Hz, 1H), 7.70 (d, *J* 3.0 Hz, 1H), 8.18 (d, *J* 3.0 Hz, 1H), 8.32 (d, *J* 3.0 Hz, 1H), 8.43 (s, 1H), 12.62 (s, 1H); δ_C_ (75 MHz, DMSO-*d*_6_) 29.7, 30.0, 32.0, 35.1, 44.8, 46.6, 71.0, 107.3, 108.6, 116.4, 118.8, 123.1, 123.9, 128.2, 129.5, 138.3, 140.1, 143.3, 145.5, 158.3, 160.6, 161.9; *m/z* (100, MH^+^); HRMS (ES): MH^+^, found 693.0359. C_22_H_21_N_4_O_2_^79^Br_4_^+^ requires 693.0358.

### 3.4. Typical Procedure for the Suzuki Cross-Coupling of **4a**–**l**

A stirred mixture of **3** (1.0 equiv.), arylboronic acid (4.5 equiv.), K_2_CO_3_ (4.0 equiv.), PdCl_2_(PPh_3_)_2_ (10% of **3**), PCy_3_ (20% of **3**) in dioxane-water (3:1, v/v) was placed in a two-necked flask with a stirrer bar, rubber septum, and a condenser. The mixture was flushed with N_2_ gas for five minutes and a balloon filled with N_2_ gas was connected on top of a condenser. The mixture was heated with stirring for 5 h and then cooled to room temperature. An ice-cold water was added to the mixture and the resulting precipitate was filtered and washed with 20% hot ethyl acetate/hexane to afford **4**. The following products were prepared in this fashion:

*2-(3-(2-Methyl-6,8-diphenyl-4-oxo-1,2,3,4-tetrahydroquinazolin-2-yl)-propyl)-6,8-diphenylquinazolin-4(3H)-ones* (**4a**). A mixture of **3a** (0.50 g, 0.75 mmol), phenylboronic acid (0.41 g, 3.39 mmol), PdCl_2_(PPh_3_)_2_ (0.05 g, 0.07 mmol), PCy_3_ (0.04 g; 0.15 mmol) and K_2_CO_3_ (0.42 g; 3.01 mmol) in dioxane-water (40 mL) afforded **4a** as a solid (0.39 g, 80%), mp. 203–206 °C; ν_max_ (neat) cm^−1^ 3389, 3178, 1673, 1614, 1560, 1488, 1445, 1364, 1344, 1259, 1225, 1133, 1017, 939, 875, 737, 681; δ_H_ (300 MHz, DMSO-*d*_6_) 1.37 (s, 3H), 1.69–1.84 (m, 4H), 2.55 (s, 2H), 5.62 (s, 1H), 7.25–7.52 (m, 15H), 7.61 (d, *J* 7.8 Hz, 2H), 7.68 (d, *J* 7.5 Hz, 2H), 7.80 (d, *J* 7.5 Hz, 2H), 7.95 (s, 1H), 8.02 (s, 1H), 8.18 (s, 1H), 8.33 (s, 1H), 12.33 (s, 1H); δ_C_ (75 MHz, DMSO-*d*_6_) 27.0, 33.2 (2 × C), 39.9, 74.9, 120.5, 127.5, 128.0, 130.1, 131.2, 132.0, 132.2, 132.4, 132.7, 133.0, 133.1, 133.4, 134.1, 134.3, 134.4 (2 × C), 134.5, 134.6, 136.1, 138.3, 138.8, 142.8, 143.2, 143.8, 144.3, 144.5, 145.1, 148.5, 150.8, 162.1, 167.6, 168.3; *m/z* 653 (100, MH^+^); HRMS (ES): MH^+^, found 653.2914. C_44_H_37_N_4_O_2_^+^ requires 653.2917.

2-(3-(6,8-Bis(2-(4-fluorophenyl)-2-methyl)-4-oxo-1,2,3,4-tetrahydroquinazolin-2-yl)propyl)-6,8-bis(2-(4-fluorophenyl))quinazolin-4(3H)-one (**4b**). A mixture of **3a** (0.50 g, 0.75 mmol), 4-fluoro- phenylboronic acid (0.47 g, 3.39 mmol), PdCl_2_(PPh_3_)_2_ (0.05 g, 0.08 mmol), PCy_3_ (0.04 g; 0.15 mmol) and K_2_CO_3_ (0.42 g; 3.01 mmol) in dioxane-water (40 mL) afforded **4b** as a solid (0.44 g, 81%), mp. 241–243 °C; ν_max_ (neat) cm^−1^ 3409, 3180, 3043, 1685, 1653, 1623, 1608, 1508, 1480, 1465, 1225, 1158, 905, 824, 557; δ_H_ (300 MHz, DMSO-*d*_6_) 1.36 (s, 3H), 1.64–1.80 (m, 4H), 2.50–2.54 (m, 2H), 5.70 (s, 1H), 7.17–7.35 (m, 7H), 7.40 (d, *J* 3.0 Hz, 1H), 7.47 (dd, *J* 6.0 and 6.9 Hz, 2H), 7.62 (dd, *J* 8.4 and 4.8 Hz, 2H), 7.74 (dd, *J* 8.4 and 4.8 Hz, 2H), 7.84–7.89 (m, 4H), 8.02 (s, 1H), 8.16 (s, 1H), 8.29–8.30 (d, *J* 3.0 Hz, 1H), 12.34 (s, 1H); δ_C_ (75 MHz, DMSO-*d*_6_) 21.9, 28.3 (2 × C), 34.8, 69.9, 114.9 (d, ^2^*J*_CF_ 21.0 Hz), 115.4, 116.1 (d, ^2^*J*_CF_ 21.4 Hz), 116.3 (d, ^2^*J*_CF_ 21.1 Hz), 116.4 (d, ^2^*J*_CF_ 21.3 Hz), 122.4, 123.0, 125.1, 127.1, 128.0, 128.1 (d, ^3^*J*_CF_ 8.3 Hz), 129.5 (d, ^3^*J*_CF_ 7.9 Hz), 131.5 (d, ^3^*J*_CF_ 8.3 Hz), 133.0 (d, ^3^*J*_CF_ 7.9 Hz), 133.3, 133.7, 134.5 (d, ^4^*J*_CF_ 3.2 Hz), 135.0 (d, ^4^*J*_CF_ 3.2 Hz), 135.9 (d, ^4^*J*_CF_ 3.2 Hz), 136.6 (d, ^4^*J*_CF_ 2.9 Hz), 136.8, 138.1, 143.6, 145.6, 157.2, 160.5, 161.7 (d, ^1^*J*_CF_ 241.1 Hz), 162.2 (d, ^1^*J*_CF_ 242.2 Hz, 2 × C signals), 162.7 (d, ^1^*J*_CF_ 243.6 Hz), 162.5, 163.3; *m/z* 725 (100, MH^+^); HRMS (ES): MH^+^, found 725.2546. C_44_H_33_N_4_O_2_F_4_^+^ requires 725.2540.

2-(3-(6,8-Bis(2-(4-methoxyphenyl)-2-methyl)-4-oxo-1,2,3,4-tetrahydroquinazolin-2-yl)propyl)-6,8-bis-(2-(4-methoxyphenyl))quinazolin-4(3H)-one (**4c**). A mixture of **3a** (0.50 g, 0.75 mmol), 4-methoxyphenylboronic acid (0.52 g, 3.39 mmol), PdCl_2_(PPh_3_)_2_ (0.05 g, 0.07 mmol), PCy_3_ (0.04 g, 0.15 mmol) and K_2_CO_3_ (0.42 g, 3.01 mmol) in dioxane-water (40 mL) afforded **4c** as a solid (0.43 g, 74%), mp. 181‒184 °C; ν_max_ (neat) cm^−1^ 3376, 3195, 2933, 1668, 1607, 1513, 1462, 1379, 1287, 1254, 1177, 1028, 1109, 9345, 824, 792; δ_H_ (300 MHz, DMSO-*d*_6_) 1.32 (s, 3H), 1.62–1.78 (m, 4H), 2.46 (s, 2H), 5.47 (s, 1H), 3.72 (s, 2H), 3.73 (s, 3H), 3.78 (s, 3H), 6.90 (d, *J* 3.0 Hz, 2H), 6.90–6.96 (m, 6H), 7.03 (d, *J* 9.3 Hz, 2H), 7.34 (d, *J* 7.5 Hz, 2H), 7.48 (d, *J* 7.8 Hz, 2H), 7.61 (d, *J* 7.8 Hz, 2H), 7.71 (d, *J* 9.0 Hz, 2H), 7.80 (s, 1H), 7.92 (s, 1H), 8.10 (s, 1H), 8.20 (s, 1H), 12.24 (s, 2H); δ_C_ (75 MHz, DMSO-*d*_6_) 28.2 (2 × C), 34.8, 55.4, 55.5, 55.6, 55.7, 66.9, 69.8, 113.6, 114.8, 114.9, 115.0, 115.4, 121.7, 122.5, 124.2, 126.7, 127.3, 127.7, 127.8, 128.3 128.5, 129.0, 130.4, 130.5, 131.2, 131.9, 132.2, 132.7, 133.0, 137.5, 138.8, 143.2, 145.2, 156.5, 158.7, 159.1, 159.7, 162.7, 163.5; *m/z* 773 (100, MH^+^); HRMS (ES): MH^+^, found 773.3356. C_48_H_45_N_4_O_6_^+^ requires 773.3339.

*2-(3-(2-Ethyl-6,8-diphenyl-4-oxo-1,2,3,4-tetrahydroquinazolin-2-yl)-propyl)-6,8-diphenylquinazolin-4(3H)-one* (**4d**). A mixture of **3b** (0.50 g, 0.74 mmol), phenylboronic acid (0.41 g, 3.32 mmol), PdCl_2_(PPh_3_)_2_ (0.05 g, 0.07 mmol), PCy_3_ (0.04 g, 0.15 mmol) and K_2_CO_3_ (0.41 g, 2.95 mmol) in dioxane-water (40 mL) afforded **4d** as a solid (0.31 g, 63%), mp. 223–226 °C; ν_max_ (neat) cm^−1^ 3384, 3177, 1665, 1601, 1487, 1463, 1439, 1249, 896.2, 808, 758, 695; δ_H_ (300 MHz, DMSO-*d*_6_) 0.84 (t, *J* 7.5 Hz, 3H), 1.57–1.81 (m, 6H), 2.50–2.54 (m, 2H), 5.40 (s, 1H), 7.27–7.53 (m, 15H), 7.60 (d, *J* 7.8 Hz, 2H), 7.68 (d, *J* 7.8 Hz, 2H), 7.81 (d, *J* 7.5 Hz, 2H), 7.94 (d, *J* 3.0 Hz, 1H), 8.03 (m, 2H) 8.33 (s, 1H), 12.33 (s, 1H); δ_C_ (75 MHz, DMSO-*d*_6_) 7.9, 21.1, 30.6, 33.1, 34.5, 72.1, 114.2, 121.9, 122.4, 124.5, 125.6, 126.4, 126.8, 126.9, 127.1, 127.5 (2 × C), 127.8, 128.0, 128.7, 128.8, 129.0, 129.1, 130.5, 132.7, 133.2, 137.3, 137.7, 138.3, 138.7, 138.9, 139.6, 143.3, 145.2, 157.1, 162.5, 163.3; *m/z* 667 (100, MH^+^); HRMS (ES): MH^+^, found 667.3094. C_45_H_39_N_4_O_2_^+^ requires 667.3073.

2-(3-(2-Ethyl-6,8-bis(2-(4-fluorophenyl))-4-oxo-1,2,3,4-tetrahydroquinazolin-2-yl)propyl)-6,8-bis(2-(4-fluorophenyl))quinazolin-4(3H)-one (**4e**). A mixture of **3b** (0.50 g, 0.74 mmol), 4-fluorophenylboronic acid (0.46 g, 3.32 mmol), PdCl_2_(PPh_3_)_2_ (0.05 g, 0.07 mmol), PCy_3_ (0.04 g, 0.15 mmol) and K_2_CO_3_ (0.41 g, 2.95 mmol) in dioxane-water (40 mL) afforded **4e** as a solid (0.34 g, 63%), mp. 252–254 °C; ν_max_ (neat) cm^−1^ 3430, 3186, 1690, 1656, 1652, 1608, 1509, 1482, 1464, 1227, 1158, 825; δ_H_ (300 MHz, DMSO-*d*_6_) 0.81 (t, *J* 7.5 Hz, 3H), 1.52–1.77 (m, 6H), 2.48–2.52 (m, 2H), 5.47 (s, 1H), 7.13–7.33 (m, 9H), 7.44 (dd, *J* 6.0 and 6.9 Hz, 2H), 7.59 (dd, *J* 6.3 and 8.4 Hz, 2H), 7.71 (dd, *J* 6.3 and 8.4 Hz, 2H), 7.81–7.86 (m, 3H), 8.00 (s, 2H), 8.27 (s, 1H), 12.31 (s, 1H); δ_C_ (75 MHz, DMSO-*d*_6_) 8.4, 21.5, 33.7 (2 × C), 34.8, 72.7, 114.5, 114.9 (d, ^2^*J*_CF_ 21.1 Hz), 116.1 (d, ^2^*J*_CF_ 21.1 Hz), 116.4 (d, ^2^*J*_CF_ 21.4 Hz), 116.5, 122.5, 123.0, 125.0, 126.5, 127.4, 128.0 (d, ^3^*J*_CF_ 7.9 Hz), 129.5 (d,^3^*J*_CF_ 8.3 Hz), 131.5 (d, ^3^*J*_CF_ 8.3 Hz), 133.0 (d, ^3^*J*_CF_ 8.0 Hz), 133.3, 133.7, 134.5 (d, ^4^*J*_CF_ 3.1 Hz), 134.9 (d, ^4^*J*_CF_ 3.2 Hz), 135.9 (d, ^4^*J*_CF_ 3.1 Hz), 136.6 (d, ^4^*J*_CF_ 3.2 Hz), 136.8, 138.1, 144.1, 145.6, 157.2, 161.7 (d, ^1^*J*_CF_ 241.9 Hz), 162.1 (d, ^1^*J*_CF_ 242.5 Hz, 2 × C), 162.5, 163.3, 162.6 (d, ^1^*J*_CF_ 243.6 Hz); *m/z* 739 (100, MH^+^); HRMS (ES): MH^+^, found 739.2709. C_45_H_35_N_4_O_2_F_4_^+^ requires 739.2696.

2-(3-(2-Ethyl-6,8-bis(2-(4-methoxyphenyl))-4-oxo-1,2,3,4-tetrahydroquinazolin-2-yl)propyl)-6,8-bis(2-(4-methoxyphenyl))quinazolin-4(3H)-ones (**4f**). A mixture of **3b** (0.50 g, 0.74 mmol), 4-methoxy- phenylboronic acid (0.51 g, 3.32 mmol), PdCl_2_(PPh_3_)_2_ (0.05 g, 0.07 mmol), PCy_3_ (0.04 g, 0.15 mmol) and K_2_CO_3_ (0.41 g, 2.95 mmol) in dioxane-water (40 mL) afforded **4f** as a solid (0.35 g, 60%), mp. 192–195 °C; ν_max_ (neat) cm^−1^ 3387, 3175, 1668, 1607, 1513, 1484, 1464, 1286, 1246, 1180, 1028, 829; δ_H_ (300 MHz, DMSO-*d*_6_) 0.82 (t, *J* 7.8 Hz, 3H), 1.54–1.80 (m, 6H), 2.50–2.54 (m, 2H), 3.75 (s, 6H), 3.77 (s, 3H), 3.81 (s, 3H), 5.26 (s, 1H), 6.92–6.99 (m, 5H,), 7. 06 (d, *J* 9.3 Hz, 2H), 7.31 (d, *J* 3.3 Hz, 2H), 7.35 (d, *J* 9.3 Hz, 2H), 7.50 (d, *J* 9.3 Hz, 2H), 7.63 (d, *J* 9.0 Hz, 2H) 7.74 (d, *J* 9.0 Hz, 2H), 7.83 (s, 1H), 7.97 (d, *J* 9.3 Hz, 2H), 8.23 (s, 1H), 12.25 (s, 1H); δ_C_ (75 MHz, DMSO-*d*_6_) 8.5, 21.7, 33.5, 34.9, 55.5, 55.6, 55.7, 55.8, 66.9, 72.6, 113.6, 114.6, 114.8, 114.9, 115.0, 121.7, 122.5, 124.1, 126.7, 127.2, 127.7, 128.3, 128.4, 128.5, 129.4, 130.4, 130.5, 131.2, 131.9, 132.2, 132.8, 133.0, 137.5, 138.8, 143.6, 145.2, 156.5, 158.6, 159.1, 159.1, 162.7, 163.6; *m/z* 787 (100, MH^+^); HRMS (ES): MH^+^, found 787.3500. C_49_H_47_N_4_O_6_^+^ requires 737.3496.

*2-(3-(2-Methyl-6,8-diphenyl-4-oxo-1,2,3,4-tetrahydroquinazolin-2-yl)-2-methylpropyl)-6,8-diphenyl-quinazolin-4(3H)-one* (**4g**). A mixture of **3c** (0.50 g, 0.74 mmol), phenylboronic acid (0.41 g, 3.32 mmol), PdCl_2_(PPh_3_)_2_ (0.05 g, 0.07 mmol), PCy_3_ (0.04 g, 0.15 mmol) and K_2_CO_3_ (0.41 g, 2.95 mmol) in dioxane-water (40 mL) afforded **4a** as a solid (0.37 g, 75%), mp. 211-215 °C; ν_max_ (neat) cm^−1^ 3397, 3167, 1685, 1655, 1606, 1491, 1464, 1388, 1318, 1250, 94, 758, 696; δ_H_ (300 MHz, DMSO-*d*_6_) 1.00 (d, *J* 4.5 Hz, 3H), 1.35 (s, 3H), 1.69–1.82 (m, 2H), 2.32–2.55 (m, 3H), 5.62 (s, 1H), 7.26–7.53 (m, 15H), 7.61 (d, *J* 7.8 Hz, 2H), 7.68 (d, *J* 6.0 Hz, 2H), 7.79 (d, *J* 6.3, 2H), 7.96 (s, 1H), 8.01 (s, 1H), 8.16 (s, 1H), 8.32 (s, 1H), 12.26 (s, 1H); δ_C_ (75 MHz, DMSO-*d*_6_) 21.8, 28.4 (2 × C), 43.2, 46.1, 70.1, 115.7, 122.5, 123.0, 125.1, 126.2, 127.0, 127.4 (2 × C), 127.6, 128.0, 128.1, 128.3, 128.4, 129.3, 129.4 (2 × C), 129.6, 131.0, 133.2, 133.8, 137.8, 138.2, 138.9, 139.4, 139.5, 140.1, 143.4, 145.8, 156.2, 162.6, 163.5; *m/z* 667 (100, MH^+^); HRMS (ES): MH^+^, found 667.3065. C_45_H_39_N_4_O_2_^+^ requires 667.3073.

2-(3-(6,8-Bis(2-(4-fluorophenyl))-2-methyl-4-oxo-1,2,3,4-tetrahydroquinazolin-2-yl)-2-methylpropyl)-6,8-bis(2-(4-fluorophenyl))quinazolin-4(3H)-one (**4h**). A mixture of **3c** (0.50 g, 0.74 mmol), 4-fluorophenylboronic acid (0.46 g, 3.32 mmol), PdCl_2_(PPh_3_)_2_ (0.05 g, 0.07 mmol), PCy_3_ (0.04 g, 0.15 mmol) and K_2_CO_3_ (0.41 g, 2.95 mmol) in dioxane-water (40 mL) afforded **4h** as a solid (0.38 g, 70%), mp. 265–268 ºC; ν_max_ (neat) cm^−1^ 3409, 3178, 3042, 1692, 1655, 1608, 1507, 1467, 1227, 1158, 1096, 821, 558; δ_H_ (300 MHz, DMSO-*d*_6_) 0.99 (d, *J* 6.3 Hz, 3H), 1.33 (s, 3H), 1.63–1.84 (m, 2H), 2.34–2.43 (m, 3H), 5.76 (s, 1H), 7.11–7.24 (m, 5H), 7.29–7.38 (m, 3H), 7.44 (dd, *J* 6.3 and 8.4 Hz, 2H), 7.62 (dd, *J* 6.3 and 8.4 Hz, 2H), 7.73 (dd, *J* 6.0 and 9.3 Hz, 3H), 7.84 (dd, *J* 6.0 and 8.5 Hz, 2H), 7.89 (d, *J* 3.0 Hz, 1H) 8.15 (s, 1H), 8.23 (s, 1H), (s, 1H), 12.27 (s, 1H); δ_C_ (75 MHz, DMSO-*d*_6_) 22.1, 28.4 (*2 × C*), 46.6, 70.1, 114.8 (d, ^2^*J*_CF_ 21.1 Hz), 115.8, 116.1 (d, ^2^*J*_CF_ 21.4 Hz), 116.2 (d, ^2^*J*_CF_ 21.0 Hz), 116.3 (d, ^2^*J*_CF_ 21.1 Hz), 122.3, 123.3, 125.1, 127.4, 128.1 (*2 × C*), 128.2 (d, ^3^*J*_CF_ 7.7 Hz), 129.2 (d, ^3^*J*_CF_ 8.3 Hz), 131.5 (d, ^3^*J*_CF_ 7.9 Hz), 132.1, 132.9 (d, ^3^*J*_CF_ 7.9 Hz), 133.1, 134.4 (d, ^4^*J*_CF_ 2.9 Hz), 134.8, 135.8 (d, ^4^*J*_CF_ 2.9 Hz), 136.6 (d, ^4^*J*_CF_ 3.1 Hz), 136.7 (d, ^4^*J*_CF_ 3.1 Hz), 137.1, 143.6 (2 × C), 147.2, 161.8 (d, ^1^*J*_CF_ 239.6 Hz), 161.9 (d, ^1^*J*_CF_ 242.2 Hz), 162.1 (d, ^1^*J*_CF_ 241.0 Hz), 162.4 (d, ^1^*J*_CF_ 242.8 Hz), 163.4 (2 × C); *m/z* 739 (100, MH^+^); HRMS (ES): MH^+^, found 739.2703. C_45_H35N_4_O_2_F_4_^+^ requires 739.2696.

2-(3-(6,8-Bis(2-(4-methoxyphenyl))-2-methyl-4-oxo-1,2,3,4-tetrahydroquinazolin-2-yl)-2-methylpropyl)-6,8-bis(2-(4-methoxyphenyl))quinazolin-4(3H)-one (**4i**). A mixture of **3c** (0.50 g, 0.74 mmol), 4-methoxyphenylboronic acid (0.51 g, 3.32 mmol), PdCl_2_(PPh_3_)_2_ (0.05 g, 0.07 mmol), PCy_3_ (0.04 g, 0.15 mmol) and K_2_CO_3_ (0.41 g, 2.95 mmol) in dioxane-water (40 mL) afforded **4i** as a solid (0.42 g, 73%), mp. 194–196 °C; ν_max_ (neat) cm^−1^ 3395, 3158.7, 2834, 1677, 1607, 1512, 1463, 1285, 1244, 1178, 1030, 828; δ_H_ (300 MHz, DMSO-*d*_6_) 0.97 (d, *J* 6.3 Hz, 3H), 1.35 (s, 3H), 1.67–1.81 (m, 2H), 2.35–2.41 (m, 3H), 3.71 (s, 3H), 3.75 (s, 3H), 3.77 (s, 3H), 3.81 (s, 3H), 5.50 (s, 1H), 6.94 (t, *J* 7.5 Hz, 3 × 2H), 7.05 (d, *J* 9.0 Hz, 2H), 7.32–7.34 (m, 3H), 7.51 (d, *J* 7.5, 2H), 7.63 (d, *J* 7.5 Hz, 2H), 7.73 (d, *J* 7.8 Hz, 2H), 7.85 (s, 1H), 7.94 (s, 1H), 8.13 (s, 1H), 8.22 (s, 1H), 12.18 (s, 1H); δ_C_ (75 MHz, DMSO-*d*_6_) 21.8, 28.2 (2 × C), 43.1, 46.2, 55.4, 55.5, 55.6, 55.7, 70.0, 113.6, 114.8 (2 × C), 115.0, 115.7, 121.7, 122.4, 124.1, 127.3, 128.1, 128.5, 129.1, 130.3, 130.5, 131.1, 131.9, 132.2 (2 × C), 132,7, 133.0, 137.5, 138.9, 142.9, 145.2, 155.6, 158.7, 159.0 (2 × C), 159.7, 162.6, 163.7; *m/z* 787 (100, MH^+^); HRMS (ES): MH^+^, found 787.3502. C_49_H_47_N_4_O_6_^+^ requires 787.3496.

*2-(3-(2-Methyl-6,8-diphenyl-4-oxo-1,2,3,4-tetrahydroquinazolin-2-yl)-2,2-dimethylpropyl)-6,8-diphenylquinazolin-4(3H)-ones* (**4j**). A mixture of **3d** (0.50 g, 0.72 mmol), phenylboronic acid (0.40 g, 3.25 mmol), PdCl_2_(PPh_3_)_2_ (0.05 g, 0.07 mmol), PCy_3_ (0.04 g, 0.15 mmol) and K_2_CO_3_ (0.40 g, 2.89 mmol) in dioxane-water (40 mL) afforded **4j** as a solid (0.37 g, 76%), mp. 289–292 °C; ν_max_ (neat) cm^−1^ 3410, 3194, 3055, 1688, 1627, 1601, 1518, 1495, 1465, 1385, 1260, 1213, 1029, 894, 788, 762, 698; δ_H_ (300 MHz, DMSO-*d*_6_) 0.89 (s, 3H), 1.02 (s, 3H), 1.11 (s, 3H) 1.55 (d, *J* 15.0 Hz, 1H), 2.10 (d, *J* 13.8 Hz, 1H), 2.64 (d, *J* 15.3 Hz, 1H), 2.97 (d, *J* 13.5 Hz, 1H), 5.26 (s, 1H), 7.08 (t, *J* 7.8 Hz, 2H) 7.22–7.45 (m, 13H), 7.52 (t, *J* 7.5 Hz, 2H), 7.61 (d, *J* 7.8 Hz, 2H), 7.84 (d, *J* 7.8 Hz, 2H), 7.93 (d, *J* 6.0 Hz, 2H), 7.99 (s, 1H), 8.36 (s, 1H) 12.27 (s, 1H); δ_C_ (75 MHz, DMSO-*d*_6_) 29.7, 29.9, 31.0, 35.0, 45.2, 46.6, 56.6, 70.6, 115.1, 122.4, 123.1, 125.1, 126.2, 127.0, 127.2, 127.4, 127.7, 127.8, 127.9, 128.1, 128.4, 129.0, 129.1, 129.4, 129.7, 130.6, 133.2, 134.1, 137.8, 139.3, 139.4, 139.8, 140.1, 142.9, 145.4, 156.1, 162.4, 162.8; *m/z* 681 (100, MH^+^); HRMS (ES): MH^+^, found 681.3232. C_46_H_41_N_4_O_2_^+^ requires 681.3230.

2-(3-(6,8-Bis(2(4-fluorophenyl))-2-methyl-4-oxo-1,2,3,4-tetrahydroquinazolin-2-yl)-2,2-dimethylpropyl)-6,8-bis(2-(4-fluorophenyl))quinazolin-4(3H)-one (**4k**). A mixture of **3d** (0.50 g, 0.72 mmol), 4-fluorophenylboronic acid (0.46 g, 3.25 mmol), PdCl_2_(PPh_3_)_2_ (0.05 g, 0.07 mmol), PCy_3_ (0.04 g, 0.15 mmol) and K_2_CO_3_ (0.40 g, 2.89 mmol) in dioxane-water (40 mL) afforded **4k** as a solid (0.42 g, 77%), mp. 303–305 °C; ν_max_ (neat) cm^−1^ 3387, 3181, 3048, 1688, 1653, 1608, 1507, 1466, 1381, 1222, 1158, 1015, 825, 804, 791, 636, 565, 517; δ_H_ (300 MHz, DMSO-*d*_6_) 1.01 (s, 3H), 1.05 (s, 3H), 1.07 (s, 3H) 1.59 (d, *J* 15.3 Hz, 1H), 2.06 (d, *J* 15.3 Hz, 1H), 2.67 (d, *J* 13.8 Hz, 1H), 2.85 (d, *J* 15.3 Hz, 1H), 5.39 (s, 1H), 7.05 (t, *J* 9.0 Hz, 2H), 7.17 (t, *J* 9.0 Hz, 2H), 7.20 (t, *J* 9.0 Hz, 2H), 7.30–7.39 (m, 5H), 7.50 (t, *J* 6.3 Hz, 2H), 7.62 (t, *J* 6.3 Hz, 2H), 7.85–7.88 (m, 3H), 7.93 (s, 1H), 8.02 (br s, 1H), 8.32 (s, 1H), 12.27 (s, 1H); δ_C_ (75 MHz, DMSO-*d*_6_) 29.7, 30.0, 30.9, 35.0, 45.4, 47.0, 70.5, 115.0, 115.2 (d, ^2^*J*_CF_ 20.5 Hz), 116.0 (d, ^2^*J*_CF_ 21.1 Hz), 116.1 (d, ^2^*J*_CF_ 21.4 Hz), 116.4 (d, ^2^*J*_CF_ 21.4 Hz), 122.3, 123.1, 125.0, 127.1, 128.0, 128.1 (d, ^3^*J*_CF_ 8.0 Hz), 129.5 (d, ^3^*J*_CF_ 8.0 Hz), 131.2 (d, ^3^*J*_CF_ 8.3 Hz), 132.7 (d, ^3^*J*_CF_ 8.0 Hz), 133.2, 134.0, 134.1 (d, ^4^*J*_CF_ 3.2 Hz), 135.4 (d, ^4^*J*_CF_ 3.2 Hz), 135.9 (d, ^4^*J*_CF_ 2.9 Hz), 136.5 (d, ^4^*J*_CF_ 2.9 Hz), 136.9, 138.5, 143.0, 145.3, 156.4, 161.8 (d, ^1^*J*_CF_ 241.7 Hz), 162.0 (d, ^1^*J*_CF_ 243.0 Hz), 162.1 (d, ^1^*J*_CF_ 242.4 Hz), 162.6 (d, ^1^*J*_CF_ 243.5 Hz), 162.3, 162.6; *m/z* 739 (100, MH^+^); HRMS (ES): MH^+^, found 739.2709. C_45_H_35_N_4_O_2_F_4_^+^ requires 739.2696.

2-(3-(6,8-Bis(2-(4-methoxyphenyl))-2-methyl-4-oxo-1,2,3,4-tetrahydroquinazolin-2-yl)-2,2-dimethylpropyl)-6,8-bis(2-(4-methoxyphenyl))quinazolin-4(3H)-one (**4l**). A mixture of **3d** (0.50 g,0.72 mmol), 4-methoxyphenylboronic acid (0.49 g, 3.25 mmol), PdCl_2_(PPh_3_)_2_ (0.05 g, 0.07 mmol), PCy_3_ (0.04 g, 0.15 mmol) and K_2_CO_3_ (0.40 g, 2.89 mmol) in dioxane-water (40 mL) afforded **4l** as a solid (0.47 g, 81%), mp. 228–230 °C; ν_max_ (neat) cm^−1^ 3177, 3037, 2834, 1677, 1607, 1511, 1463, 1386, 1244, 1178, 1034, 899, 827; δ_H_ (300 MHz, DMSO-*d*_6_) 0.93 (s, 3H), 1.03 (s, 3H), 1.08 (s, 3H) 1.57 (d, *J* 15.3 Hz, 1H), 2.06 (d, *J* 13.8 Hz, 1H), 2.67 (d, *J* 13.8 Hz, 1H), 2.94 (d, *J* 13.8 Hz, 1H), 3.56 (s, 3H), 3.69 (s, 3H), 3.77 (s, 3H), 3.82 (s, 3H), 5.27 (s, 1H), 6.79 (d, *J* 9.0 Hz, 2H) 6.88 (d, *J* 9.3 Hz, 2H), 6.96 (d, J 9.3 Hz, 2H), 7.07 (d, *J* 9.3 Hz, 2H), 7.31 (d, *J* 7.8 Hz, 2x2H), 7.52 (d, *J* 9.0 Hz, 2H), 7.75 (d, *J* 9.0 Hz, 2 × 2H), 7.85 (d, *J* 3.3 Hz, 2H), 7.95 (s, 1H), 12.22 (s, 1H); δ_C_ (75 MHz, DMSO-*d*_6_) 29.8, 30.0, 30.7, 35.1, 45.2, 46.7, 55.4, 55.5, 55.6, 55.7, 70.5, 113.9, 114.5, 114.8, 115.0, 121.8, 122.2, 124.1, 126.7, 127.3, 127.7, 127.9, 128.3, 128.5, 128.9, 129.4, 130.1, 130.3, 131.6, 131.8, 132.7, 133.5, 137.5, 139.4, 142.6, 145.0, 155.6, 158.7, 159.0, 159.7, 162.5, 163.0; *m/z* 801 (100, MH^+^); HRMS (ES): MH^+^, found 801.3665. C_50_H_49_N_4_O_6_^+^ requires 801.3652.

## 4. Conclusions

Elaboration of the 2-(3-(2-alkyl-6,8-dibromo-4-oxo-1,2,3,4-tetrahydroquinazolin-2-yl)propyl)-6,8-dibromoquinazolin-4(3*H*)-one scaffold via Suzuki-Miyaura cross-coupling with arylboronic acids afforded novel 2-(3-(2-alkyl-6,8-diaryl-4-oxo-1,2,3,4-tetrahydroquinazolin-2-yl)propyl)-6,8-diaryl-quinazolin-4(3*H*)-ones. The electronic absorption and emission properties of these bisquinazolinone derivatives showed a strong correlation with the substituents on the aryl groups and the size of the alkyl substituent on the 2-position of the quinazolin-4(1*H*)-one moiety as well as the substitution pattern on the propyl chain. The solvent-dependent emission characteristics of the polyaryl bisquinazolinones **4**, which result from the dipolar interaction with the polar solvent suggest the ICT character of the emission state. Based on the orbital diagrams, the electronic transitions of compounds **4** can be attributed to ICT from the aryl substituents to the quinazolinone moieties. However, due to the low relative fluorescent quantum yields in the region of λ_em_ 435 and 505 nm these compounds do not represent potential fluorophores. Chemical modification of the conjugated backbones, on the other hand, allows efficient manipulation of physical properties that are important for determining the characteristics of light emission, namely the band gap and the electronic behavior. Research is currently underway in our laboratory to combine the two aryl-substituted quinazolinone moieties directly and through a π-conjugated bridge to broaden the absorption and emission windows.
